# Primary and Secondary Cardiovascular and Kidney Prevention With Canagliflozin: Insights From the CANVAS Program and CREDENCE Trial

**DOI:** 10.1161/JAHA.123.031586

**Published:** 2024-01-19

**Authors:** Abhinav Sharma, Amir Razaghizad, Abdulaziz Joury, Adeera Levin, Harpreet S. Bajaj, G. B. John Mancini, Norman C. Wong, April Slee, Fernando G. Ang, Wally Rapattoni, Brendon L. Neuen, Clare Arnott, Vlado Perkovic, Kenneth W. Mahaffey

**Affiliations:** ^1^ Centre for Outcomes Research and Evaluation Research Institute of the McGill University Health Centre Montreal QC Canada; ^2^ Division of Cardiology McGill University Health Centre, McGill University Montreal QC Canada; ^3^ DREAM‐CV Laboratory McGill University Health Centre, McGill University Montreal QC Canada; ^4^ King Salman Heart Center, King Fahad Medical City Riyadh Saudi Arabia; ^5^ Division of Nephrology University of British Columbia Vancouver BC Canada; ^6^ LMC Healthcare Brampton ON Canada; ^7^ Centre for Cardiovascular Innovation University of British Columbia Vancouver BC Canada; ^8^ University of Calgary AB Canada; ^9^ New Arch Consulting Seattle WA; ^10^ Janssen, Inc. Toronto ON Canada; ^11^ The George Institute for Global Health, UNSW Sydney Sydney Australia; ^12^ Royal North Shore Hospital Sydney Australia; ^13^ Faculty of Medicine, UNSW Sydney Sydney Australia; ^14^ Department of Cardiology Royal Prince Alfred Hospital Sydney Australia; ^15^ Sydney Medical School University of Sydney Australia; ^16^ Stanford Center for Clinical Research, Department of Medicine Stanford University School of Medicine Stanford CA

**Keywords:** canagliflozin, primary and secondary prevention, type 2 diabetes, Diabetes, Type 2, Cardiovascular Disease, Primary Prevention, Secondary Prevention

## Abstract

**Background:**

This study evaluated the effects of canagliflozin in patients with type 2 diabetes with and without prevalent cardiovascular disease (secondary and primary prevention).

**Methods and Results:**

This was a pooled participant‐level analysis of the CANVAS (Canagliflozin Cardiovascular Assessment Study) Program and CREDENCE (Canagliflozin and Renal Events in Diabetes With Established Nephropathy Clinical Evaluation) trial. The CANVAS Program included participants with type 2 diabetes at elevated cardiovascular risk, whereas the CREDENCE trial included participants with type 2 diabetes and albuminuric chronic kidney disease. Hazard ratios (HRs) with interaction terms were obtained from Cox regression models to estimate relative risk reduction with canagliflozin versus placebo across the primary and secondary prevention groups. We analyzed 5616 (38.9%) and 8804 (61.1%) individuals in the primary and secondary prevention subgroups, respectively. Primary versus secondary prevention participants were on average younger (62.2 versus 63.8 years of age) and more often women (42% versus 31%). Canagliflozin reduced the risk of major adverse cardiovascular events (HR, 0.84 [95% CI, 0.76–0.94]) consistently across primary and secondary prevention subgroups (*P*_interaction_=0.86). Similarly, no treatment effect heterogeneity was observed with canagliflozin for hospitalization for heart failure, cardiovascular death, end‐stage kidney disease, or all‐cause mortality (all *P*_interaction_>0.5).

**Conclusions:**

Canagliflozin reduced cardiovascular and kidney outcomes with no statistical evidence of heterogeneity for the treatment effect across the primary and secondary prevention subgroups in the CANVAS Program and CREDENCE trial. Although studies on the optimal implementation of canagliflozin within these populations are warranted, these results reinforce canagliflozin's role in cardiorenal prevention and treatment in individuals with type 2 diabetes.

**Registration:**

URL: https://www.clinicaltrials.gov; Unique identifiers: NCT01032629, NCT01989754, NCT02065791.

**Nonstandard Abbreviations and Acronyms**
CANVAS: Canagliflozin Cardiovascular Assessment Study
CREDENCE: Canagliflozin and Renal Events in Diabetes With Established Nephropathy Clinical Evaluation
DECLARE‐TIMI 58: Multicenter Trial to Evaluate the Effect of Dapagliflozin on the Incidence of Cardiovascular Events
dSCr: doubling of serum creatinine
ESKD: end‐stage kidney disease
HHF: hospitalization for heart failure
MACE‐3P: 3‐point major adverse cardiovascular event
SCORED: Cardiovascular and Renal Events in Patients With Type 2 Diabetes and Moderate Renal Impairment Who Are at Cardiovascular Risk
SGLT2i: sodium‐glucose cotransporter 2 inhibitor
T2D: type 2 diabetes
UACR: urine albumin/creatinine ratio

**Clinical Perspective**

**What Is New?**

- This study evaluated the effects of canagliflozin, an SGLT2i (sodium‐glucose cotransporter 2 inhibitor) used to treat type 2 diabetes in patients with and without prevalent cardiovascular disease.
- The study did not detect differences in the treatment effects of canagliflozin between individuals with type 2 diabetes and prevalent cardiovascular disease and those without cardiovascular disease.

**What Are the Clinical Implications?**

- Canagliflozin should be considered as a valuable option for cardiovascular and renal prevention and treatment in individuals with type 2 diabetes, irrespective of their cardiovascular disease status.
- These findings emphasize the need for optimizing the use of canagliflozin in both primary and secondary prevention populations, and further research is warranted to determine the best strategies for implementing this medication effectively.

People living with type 2 diabetes (T2D) are at significantly increased risk for adverse cardiovascular events, including myocardial infarction, stroke, and heart failure (HF).^1^ Furthermore, T2D is one of the most significant risk factors for the development of chronic kidney disease (CKD) and progression to kidney failure.^2^ Prevention of cardiovascular and kidney events in individuals with T2D remains a public health priority; global population‐level prevalence studies have identified that two‐thirds of all individuals with T2D do not have overt cardiovascular disease^3^; therefore, prevention of cardiovascular and kidney disease will have significant health and economic benefits at a population level.^4^

Randomized trials evaluating SGLT2i (sodium‐glucose cotransporter 2 inhibitor) in participants with T2D at high cardiovascular risk have demonstrated substantial reductions in the risk of cardiovascular and kidney events.^5^, ^6^, ^7^, ^8^, ^9^ In the CANVAS (Canagliflozin Cardiovascular Assessment Study) Program, canagliflozin, an SGLT2i, significantly reduced the risk of major adverse cardiovascular events, defined as the composite of cardiovascular death, nonfatal myocardial infarction, or nonfatal stroke (ie, 3‐point major adverse cardiovascular event [MACE‐3P]), in participants with T2D and elevated cardiovascular risk.^10^ Furthermore, canagliflozin demonstrated significant reductions in the risk of kidney failure, MACE‐3P, and HF events in participants in the CREDENCE (Canagliflozin and Renal Events in Diabetes With Established Nephropathy Clinical Evaluation) trial.^11^ Despite this evidence, use of SGLT2i among eligible people with diabetes remains low.^12^, ^13^

Prior studies have demonstrated substantial cardiovascular and kidney benefits of SGLT2i in people with established cardiovascular disease (secondary prevention).^14^ Yet, there has been less attention paid to the prevention of cardiovascular and kidney events in individuals with T2D without overt cardiovascular disease (primary prevention). Given the substantial global proportion of individuals with T2D who do not have established cardiovascular disease, it is important to understand the impact of SGLT2i across both primary and secondary prevention populations. The evaluation of outcomes within the primary and secondary prevention subgroups was prespecified in the CANVAS Program.^14^, ^15^ In the CREDENCE trial, analysis of the primary and secondary outcomes was planned for hierarchical testing, with the analyses for the primary outcome prespecified in both subgroups. In this analysis, we evaluated the efficacy of canagliflozin for primary and secondary prevention subgroups from pooled data across the CANVAS Program and CREDENCE trial.

## METHODS

The data sharing policy of Janssen Pharmaceutical Companies of Johnson & Johnson is available at https://www.janssen.com/clinicaltrials/transparency. As noted on this site, requests for access to the study data can be submitted through the Yale Open Data Access Project site at http://yoda.yale.edu

### Data Source

In the present analysis, we conducted an individual participant data analysis of the CANVAS Program and CREDENCE trial. The designs of the CANVAS Program (CANVAS and CANVAS‐R) and the CREDENCE trial, including their setting, locations, relevant dates, periods of recruitment, and data collection practices, have previously been published in detail.^10^, ^11^, ^16^ To summarize, the CANVAS Program included 2 randomized, double‐blind, placebo‐controlled trials of canagliflozin in participants with T2D at elevated cardiovascular risk. The CREDENCE trial was a randomized, double‐blind, placebo‐controlled trial enrolling participants with T2D and albuminuric CKD. Both the CANVAS Program and CREDENCE trial included participants with and without cardiovascular disease (primary and secondary prevention populations, respectively).

### Participants and Definition of Primary and Secondary Prevention Subgroups

The CANVAS Program enrolled individuals with T2D and an estimated glomerular filtration rate (eGFR) >30 mL/min per 1.73 m^2^. Participants were either ≥30 years of age with a history of symptomatic atherosclerotic cardiovascular events (ASCVD; defined as stroke, myocardial infarction, hospitalization for unstable angina, coronary artery bypass grafting, percutaneous coronary intervention, peripheral revascularization [surgical or percutaneous], and symptomatic with documented hemodynamically significant carotid or peripheral vascular disease or amputation secondary to vascular disease [secondary prevention subgroups]) or ≥50 years of age with no prior cardiovascular events but with ≥2 of the following cardiovascular risk factors: duration of diabetes ≥10 years, systolic blood pressure >140 mm Hg on ≥1 antihypertensive agent, current smoker, microalbuminuria or macroalbuminuria, or high‐density lipoprotein cholesterol <1 mmol/L (primary prevention subgroups).

The CREDENCE trial enrolled individuals with both T2D and CKD, which was defined by an eGFR between 30 and 90 mL/min per 1.73 m^2^ with a urine albumin/creatinine ratio (UACR) of 300 to 5000 mg/g (33.9–565.6 mg/mmol). The definition of primary and secondary prevention subgroups was the same as in the CANVAS Program. All participants in the CREDENCE trial were ≥30 years of age and were receiving a stable dose of an angiotensin receptor blocker or angiotensin‐converting enzyme inhibitor for 4 weeks before randomization.

For both trials, the protocols were approved by the ethics committees at each site, and the study was conducted in accordance with the ethical principles of the Declaration of Helsinki. All participants provided written informed consent. F.G.A. had full access to all of the data in the study and takes responsibility for their integrity and the data analysis.

### Details on Randomization, Treatment, and Follow‐Up

Across studies, randomization was performed following a 2‐week, single‐blinded, run‐in period. In the CANVAS Program, participants were assigned a 1:1:1 randomization to canagliflozin 300 mg, canagliflozin 100 mg, or matched placebo. In CANVAS‐R, participants were randomized 1:1 to canagliflozin 100 mg or placebo, with an option to increase to 300 mg at week 13. CREDENCE trial participants were randomized 1:1 to receive canagliflozin 100 mg or matched placebo. Other therapy for glycemic control and risk management was continued or instituted as per best practice in line with guideline‐based care. All participants receiving any of the above dosing regimens were included in this subgroup analysis. All participants, investigators, and care providers were blinded to the study drug throughout the trial periods.

### Outcomes of Interest

The primary outcome for the CANVAS Program was MACE‐3P. For the CREDENCE trial, the primary outcome was the composite of end‐stage kidney failure (dialysis for ≥30 days, kidney transplantation, or eGFR <15 mL/min per 1.73 m^2^), doubling of serum creatinine (dSCr), or death because of kidney or cardiovascular disease. In the present post hoc analysis, the outcomes of interest were MACE‐3P, hospitalization for HF (HHF), cardiovascular death or HHF, all‐cause mortality, and kidney outcomes including progression to end‐stage kidney disease (ESKD), dSCr, and the composite of time to first ESKD or dSCr.

### Statistical Analysis

Post hoc analyses were based upon the integrated data sets from the CANVAS Program and the CREDENCE trial, and subjects were analyzed according to randomization assignment (intention to treat, canagliflozin versus placebo). Analyses are restricted to subjects with nonmissing values of imbalanced covariates (duration of diabetes, sex, baseline eGFR, and baseline UACR). Baseline characteristics were compared between participants in the primary and secondary prevention groups using the *t* test or Wilcoxon rank sum test for continuous variables and the χ^2^ test or Fisher exact test, as appropriate, for categorical variables. Categorical variables are presented as participant numbers and percentages. Continuous variables are presented as means (SDs) or medians (interquartile ranges).

Hazard ratios (HRs) and 95% CIs were calculated for participants assigned to canagliflozin versus participants assigned to placebo separately for the primary and secondary prevention groups. Outcomes were analyzed using Cox proportional hazards regression models, with treatment as the exploratory variable and adjusted for duration of diabetes ≤13 versus >13 years (the median in this sample), sex, baseline eGFR <60 versus ≥60 mL/min per 1.73 m^2^, and baseline UACR <300 versus ≥300 mg/g (33.9 mg/mmol). The selection of the adjustment variables and eGFR and UACR thresholds were determined based on clinical significance. Adjustment was required to address confounding in the primary and secondary prevention groups due to inherent differences between the CANVAS Program and CREDENCE trial. The proportional hazards assumption was assessed by including the log‐time‐by‐treatment interaction in the proportional hazards models, inspection of Kaplan‐Meier curves, and inspection of Schoenfeld residuals. Homogeneity of treatment effects across the primary and secondary prevention groups was examined by adding the main effect of the prevention group and the treatment‐by‐prevention group interaction to these models. Event rates for key outcomes of interest per 1000 participants through 2.5 years of follow‐up were estimated using Poisson regression, with adjustments for the same covariates using the same functional form as described for the proportional hazards models. The adjusted event rate serves to estimate the hypothetical rate if the entire population shared the same probability distribution of the specified covariates in the overall cohort (Table S1). More granular estimates are additionally available in Table S2. This standardization of rates, based on covariate distribution, facilitates comparisons that considers relevant covariates. Absolute risk reductions and 95% CIs between treatment groups were obtained using the delta method after postestimation from the Poisson regression model. Analyses were undertaken using SAS version 9.4.

## RESULTS

### Baseline Demographics

Overall, 14 543 participants were considered for inclusion, of which 14 420 (99.2%) were included in the present analysis (Table). The mean follow‐up time was 3.28 years. There were 5616 (38.9%) participants classified in the primary prevention subgroup and 8804 (61.1%) classified in secondary prevention subgroup. On average, the primary prevention subgroup, compared with the secondary prevention subgroup, was younger (62.2 versus 63.8 years of age), more often women (41.8% versus 31.1%), and had longer duration of diabetes (14.6 versus 14.0 years). Primary and secondary prevention participants had similar glycated hemoglobin (8.3% versus 8.3%) and eGFR (70.0 versus 70.5 mL/min per 1.73 m^2^). Primary prevention participants had higher median UACR compared with secondary prevention participants (65.5 mg/g versus 24.8 mg/g [7.4 versus 2.8 mg/mmol]). Participants in the primary versus secondary prevention group had lower use of common cardiac medications, including statins (63.5% versus 79.3%), β‐blockers (31.7% versus 60.8%), and antithrombotic agents (46.6% versus 84.0%), as well as insulin (54.0% versus 55.6%), but higher use of sulfonylurea (40.3% versus 37.8%).

**Table 1:** Baseline Characteristics of Primary and Secondary Prevention Subgroups From the CANVAS Program and CREDENCE Trial

Characteristic	Primary prevention			*P* value	Secondary prevention			*P* value
	Canagliflozin (n=3105)	Placebo (n=2511)	Total (N=5616)		Canagliflozin (n=4830)	Placebo (n=3974)	Total (N=8804)	
Age, y, mean±SD	62.1±8.3	62.4±8.3	62.2±8.3	0.346	63.7±8.7	64.0±8.7	63.8±8.7	0.102
Sex, n (%)								
Women	1311 (42.2%)	1038 (41.3%)	2349 (41.8%)	0.504	1470 (30.4%)	1264 (31.8%)	2734 (31.1%)	0.166
Men	1794 (57.8%)	1473 (58.7%)	3267 (58.2%)		3360 (69.6%)	2710 (68.2%)	6070 (68.9%)	
Race, n (%)								
White	2191 (70.6%)	1743 (69.4%)	3934 (70.0%)	0.315	3749 (77.6%)	3082 (77.6%)	6831 (77.6%)	0.739
Asian	575 (18.5%)	462 (18.4)	1037 (8.5%)		626 (13.0%)	496 (12.5%)	1122 (12.7%)	
Black	116 (3.7%)	118(4.7%)	234 (4.2%)		168 (3.5%)	12 (3.8%)	320 (3.6%)	
Other*	223 (7.2%)	188 (7.5%)	411 (7.3%)		287 (5.9%)	244 (6.1%)	531 (6.0%)	
Current smoker, n (%)	660 (21.3%)	503 (20.0%)	1163 (20.7%)	0.260	687 (14.2%)	564 (14.2%)	1251 (14.2%)	0.970
Duration of diabetes, y, mean±SD	14.5±7.2	14.8±7.4	14.6±7.3	0.155	13.7±8.5	14.3±8.6	14.0±8.6	0.003
History of hypertension, n (%)	2880 (92.8%)	2347 (93.5%)	5227 (93.1%)	0.294	4379 (90.7%)	3663 (92.2%)	8042 (91.3%)	0.012
History of heart failure, n (%)	202 (6.5%)	195 (7.8%)	397 (7.1%)	0.067	914 (18.9%)	773 (19.5%)	1687 (19.2%)	0.531
Drug therapy, n (%)								
Insulin	1648 (53.1%)	1383 (55.1%)	3031 (54.0%)	0.134	2669 (55.3%)	2224 (56.0%)	4893 (55.6%)	0.508
Sulfonylurea	1283 (41.3%)	979 (39.0%)	2262 (40.3%)	0.076	1836 (38.0%)	1491 (37.5%)	3327 (37.8%)	0.635
Metformin	2331 (75.1%)	1830 (72.9%)	4161 (74.1%)	0.062	3343 (69.2%)	2764 (69.6%)	6107 (69.4%)	0.731
GLP‐1 receptor agonist	125 (4.0%)	123 (4.9%)	248 (4.4%)	0.113	183 (3.8%)	154 (3.9%)	337 (3.8%)	0.834
DPP‐4 inhibitor	492 (15.8%)	422 (16.8%)	914 (16.3%)	0.332	572 (11.8%)	509 (12.8%)	1081 (12.3%)	0.170
Statin	1979 (63.7%)	1588 (63.2%)	3567 (63.5%)	0.702	3844 (79.6%)	3140 (79.0%)	6984 (79.3%)	0.509
Antithrombotic	1435 (46.2%)	1180 (47.0%)	2615 (46.6%)	0.561	4098 (84.8%)	3301 (83.1%)	7399 (84.0%)	0.023
RAAS inhibitor	2716 (87.5%)	2224 (88.6%)	4940 (88.0%)	0.208	4077 (84.4%)	3388 (85.3%)	7465 (84.8%)	0.272
β‐Blocker	977 (31.5%)	803 (32.0%)	1780 (31.7%)	0.681	2917 (60.4%)	2435 (61.3%)	5352 (60.8%)	0.400
Diuretic	1337 (43.1%)	1113 (44.3%)	2450 (43.6%)	0.342	2195 (45.4%)	1840 (46.3%)	4035 (45.8%)	0.423
Microvascular disease history, n (%)								
Retinopathy	799 (25.7%)	702 (28.0%)	1501 (26.7%)	0.061	1321 (27.3%)	1160 (29.2%)	2481 (28.2%)	0.056
Nephropathy	1446 (46.6%)	1376 (54.8%)	2822 (50.2%)	<0.001	1737 (36.0%)	1594 (40.1%)	3331 (37.8%)	<0.001
Neuropathy	977 (31.5%)	857 (34.1%)	1834 (32.7%)	0.034	1865 (38.6%)	1511 (38.0%)	3376 (38.3%)	0.571
History of amputation, n (%)	17 (0.5%)	3 (0.1%)	20 (0.4%)	0.007	237 (4.9%)	212 (5.3%)	449 (5.1%)	0.364
BMI, kg/m^2^, mean±SD	31.8±6.2	31.9±6.3	31.8±6.3	0.716	31.8±5.8	31.7±5.9	31.7±5.8	0.496
HbA1c, %, mean±SD	8.3±1.1	8.3±1.1	8.3±1.1	0.544	8.3±1.0	8.2±1.1	8.3±1.0	0.604
BP, mm Hg, mean±SD	139.6±15.2	139.8±15.0	139.7±15.1	0.579	136.0±16.0	136.9±16.2	136.4±16.1	0.009
DBP, mm Hg, mean±SD	79.3±9.4	79.1±9.5	79.2±9.4	0.585	76.8±9.5	77.3±9.6	77.0±9.5	0.035
Total cholesterol, mmol/L, mean±SD	4.6±1.2	4.6±1.2	4.6±1.2	0.846	4.4±1.2	4.4±1.2	4.4±1.2	0.204
Total triglycerides, mmol/L, mean±SD	2.1±1.3	2.1±1.5	2.1±1.4	0.583	2.1±1.5	2.1±1.6	2.1±1.5	0.730
HDL‐C, mmol/L, mean±SD	1.2±0.4	1.2±0.3	1.2±0.3	0.407	1.2±0.3	1.2±0.3	1.2±0.3	0.943
LDL‐C, mmol/L, mean±SD	2.4±1.0	2.4±1.0	2.4±1.0	0.801	2.3±1.0	2.3±1.0	2.3±1.0	0.215
eGFR, mL/min per 1.73 m^2^, mean±SD	71.0±21.9	68.6±22.3	70.0±22.1	<0.001	71.1±21.6	69.8±22.1	70.5±21.9	0.006
eGFR categories, mL/min per 1.73 m^2^, n (%)								
≤15	2 (0.1%)	0 (0.0%)	2 (<0.1%)	<0.001	1 (<0.1%)	1 (<0.1%)	2 (<0.1%)	0.012
≥15 to <30	46 (1.5%)	51 (2.0%)	97 (1.7%)		46 (1.0%)	55 (1.4%)	101 (1.1%)	
≥30 to <45	368 (11.9%)	333 (13.3%)	701 (12.5%)		511 (10.6%)	499 (12.6%)	1010 (11.5%)	
≥45 to <60	520 (16.7%)	523 (20.8%)	1043 (18.6%)		911 (18.9%)	777 (19.6%)	1688 (19.2%)	
≥60 to <90	1567 (50.5%)	1169 (46.6%)	2736 (48.7%)		2448 (50.7%)	1927 (48.5%)	4375 (49.7%)	
≥90	602 (19.4%)	435 (17.3%)	1037 (18.5%)		913 (18.9%)	715 (18.0%)	1628 (18.5%)	
Median (IQR) UACR	4.8	12	7.4	<0.001	2.5	3.3	2.8	<0.001
mg/mmol	1.0–65.9	1.2–96.5	1.1–78.6		0.9–37.7	0.9–57.3	0.9–46.4	
mg/g	42.2	105.9	65.5	<0.001	22.4	29	24.8	<0.001
UACR categories	8.9, 583.0	10.2, 853.0	9.4, 695.0		7.8, 333.0	8.2, 507.0	8.0, 410.0	
<30 mg/g (<3.39 mg/mmol)	1427 (46.0%)	994 (39.6%)	2421 (43.1%)	<0.001	2595 (53.7%)	2013 (50.7%)	4608 (52.3%)	<0.001
30–300 mg/g (3.39 to 33.9 mg/mmol)	583 (18.8%)	467 (18.6%)	1050 (18.7%)		990 (20.5%)	719 (18.1%)	1709 (19.4%)	
>300–≤3000 mg/g (>33.9–≤339 mg/mmol)	971 (31.3%)	916 (36.5%)	1887 (33.6%)		1107 (22.9%)	1078 (27.1%)	2185 (24.8%)	
>3000 mg/g (>339 mg/mmol)	124 (4.0%)	134 (5.3%)	258 (4.6%)		138 (2.9%)	164 (4.1%)	302 (3.4%)	

BMI indicates body mass index; CANVAS, Canagliflozin Cardiovascular Assessment Study; CREDENCE, Canagliflozin and Renal Events in Diabetes With Established Nephropathy Clinical Evaluation; DBP, diastolic blood pressure; DPP‐4, dipeptidyl peptidase 4; eGFR, estimated glomerular filtration rate; GLP‐1, glucagon‐like peptide 1; HbA1c, glycated hemoglobin; HDL‐C, high‐density lipoprotein cholesterol; IQR, interquartile range; LDL‐C, low‐density lipoprotein cholesterol; RAAS, renin‐angiotensin‐aldosterone system; SBP, systolic blood pressure; and UACR, urine albumin/creatinine ratio.

Other includes American Indian or Alaska Native, Native Hawaiian or other Pacific Islander, multiple, other, unknown, and not reported.

### Risk of MACE‐3P and HHF

**Figure 1:**
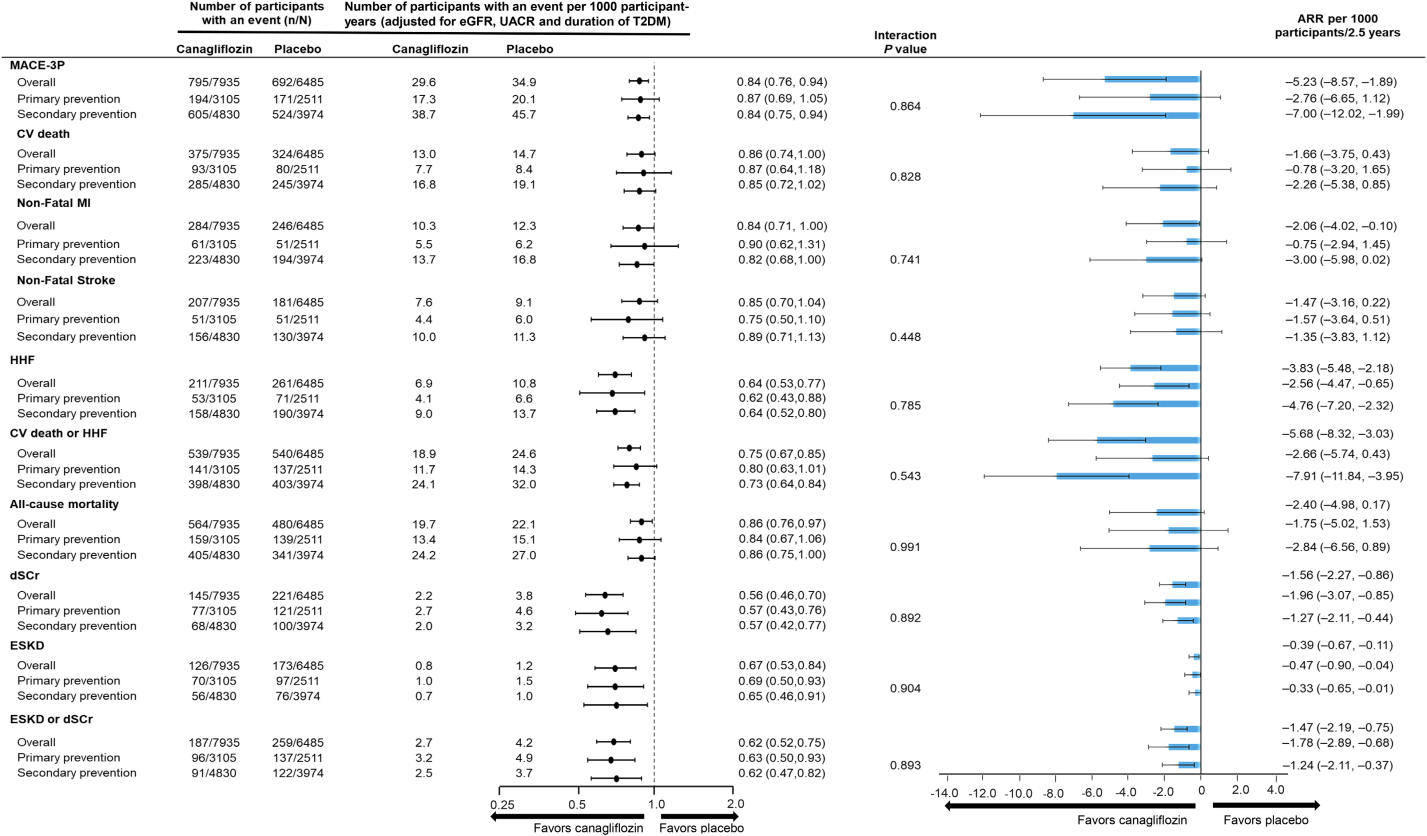
Forest plot for CV and kidney outcomes in primary and secondary prevention subgroups. Adjusted rates were calculated with Poisson regression, whereby the adjusted rate serves to estimate the hypothetical rate if the entire population shared the same probability distribution of specified covariates. Here, this comprises individuals with specific characteristics: 67.8% exhibit an eGFR >60, 67.9% possess a UACR <300, 50.6% have lived with type 2 diabetes for <13 years, and 64.8% are men. This standardization of rates, based on covariate distribution, facilitates comparisons that consider these factors. *Cox proportional hazards models and Poisson model were adjusted for diabetes duration ≤13 vs >13 years, sex, UACR <300 vs ≥300 mg/g (33.9 mg/mmol), eGFR <60 vs ≥60 mL/min per 1.73 m^2^. ARR indicates absolute risk reduction; CV, cardiovascular; dSCr, doubling of serum creatinine; eGFR, estimated glomerular filtration rate; ESKD, end‐stage kidney disease; HHF, hospitalization for heart failure; HR, hazard ratio; MACE‐3P, 3‐point major adverse cardiovascular events; MI, myocardial infarction; T2DM, type 2 diabetes mellitus; and UACR, urine albumin/creatinine ratio.

**Figure 2:**
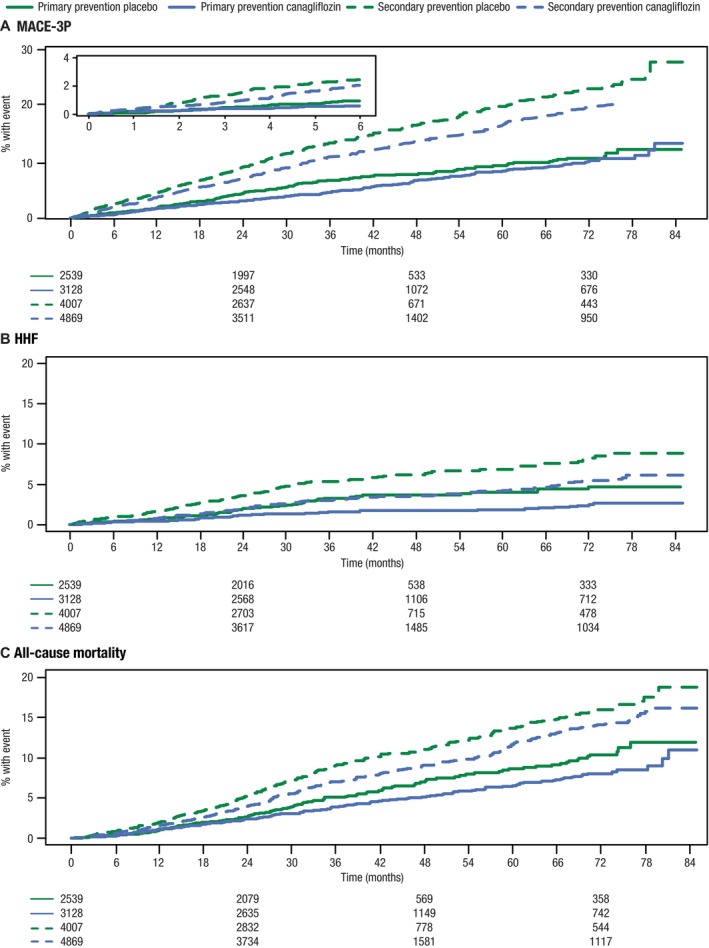
Kaplan‐Meier curves for (A) MACE, (B) HHF, and (C) all‐cause mortality. HHF indicates hospitalization for heart failure; and MACE‐3P, 3‐point major adverse cardiovascular events.

The risk of MACE‐3P and cardiovascular events was lower in the primary prevention subgroup compared with the secondary prevention subgroup as reflected by placebo group event rates (Figures 1 and 2). The adjusted placebo event rate for MACE‐3P in the primary and secondary prevention group was 20.1 per 1000 participant‐years and 45.7 per 1000 participant‐years, respectively. Overall, canagliflozin significantly reduced MACE‐3P risk (29.6 versus 34.96 per 1000 participant‐years; HR, 0.84 [95% CI, 0.76–0.94]) with no effect modification by baseline cardiovascular disease (HR, 0.87 [95% CI, 0.69–1.05] versus HR, 0.84 [95% CI, 0.75–0.94] for primary and secondary prevention subgroups, respectively; *P*_interaction_=0.864). Canagliflozin similarly reduced the risk of HHF in both the primary prevention subgroup (4.1 versus 6.6 per 1000 participant‐years; HR, 0.62 [95% CI, 0.43–0.88]) and secondary prevention subgroup (9.0 versus 13.7 per 1000 participant‐years; HR, 0.64 [95% CI, 0.52–0.80]; *P*_interaction_=0.785) with no significant treatment effect heterogeneity. For HHF, the event rate in the secondary prevention group for canagliflozin closely approximated the event rate observed in the primary prevention subgroup receiving placebo (Figure 1).

### Risk of Cardiovascular Death and Mortality Outcomes

The effect of canagliflozin on cardiovascular death (HR, 0.86 [95% CI, 0.74–1.00]) was not modified by history of cardiovascular disease (*P*_interaction_=0.828; Figures 1 and 2). Similar results were obtained for the composite end point consisting of cardiovascular death or HHF. Overall, canagliflozin significantly reduced the risk of all‐cause mortality (19.7 versus 22.1 per 1000 participant‐years; HR, 0.86 [95% CI, 0.76–0.97]; *P*=0.017) with consistent effects across subgroups (HR, 0.84 [95% CI, 0.67–1.06] and HR, 0.86 [95% CI, 0.75–1.00] for primary and secondary prevention subgroups, respectively; *P*_interaction_=0.991; Figure 1B).

### Risk of Kidney Outcomes

**Figure 3:**
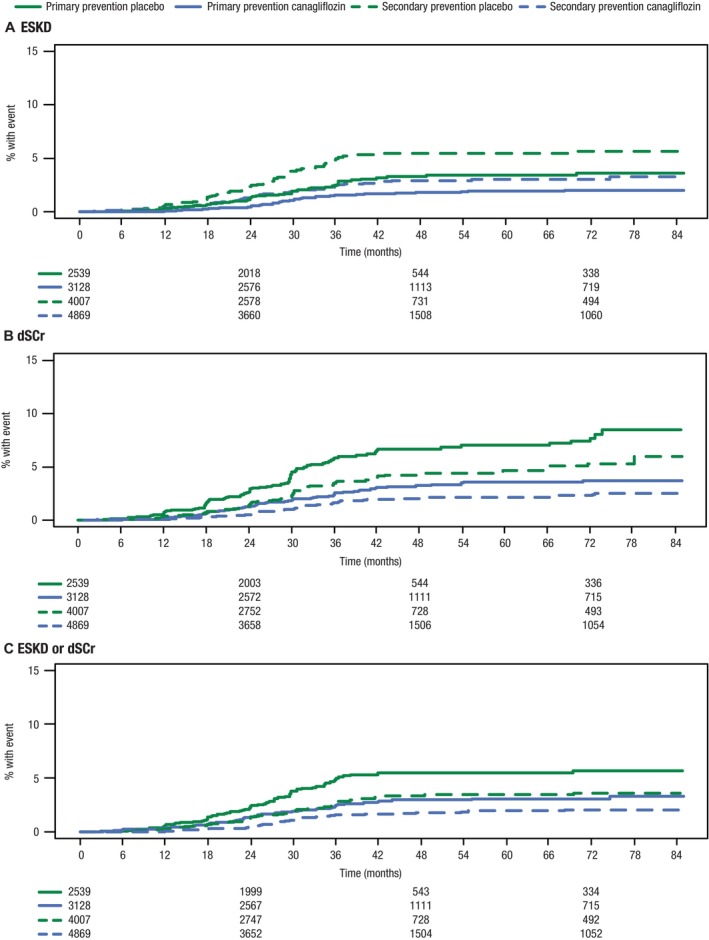
Kaplan‐Meier curves for (A) ESKD, (B) dSCr, and (C) ESKD or dSCr. dSCr indicates doubling of serum creatinine; and ESKD, end‐stage kidney disease.

Canagliflozin reduced the risk of dSCr versus placebo in both the primary prevention subgroup (2.7 versus 4.6 per 1000 participant‐years; HR, 0.57 [95% CI, 0.43–0.76]) and secondary prevention subgroup (2.0 versus 3.2 per 1000 participant‐years; HR, 0.57 [95% CI, 0.42–0.77]; *P*_interaction_=0.892; Figures 1 and 3). Canagliflozin also reduced the risk of progression to ESKD versus placebo across the primary prevention subgroup (1.0 versus 1.5 per 1000 participant‐years; HR, 0.69 [95% CI, 0.50–0.93]) and secondary prevention subgroup (0.7 versus 1.0 per 1000 participant‐years; HR, 0.65 [95% CI, 0.46–0.91]; *P*_interaction_=0.904). Similar findings were observed for the composite of ESKD and dSCr (Figures 1 and 3).

## DISCUSSION

The present study evaluated the effects of canagliflozin for primary and secondary prevention subgroups with combined participant‐level data from the randomized CANVAS Program and CREDENCE trial. We demonstrated that canagliflozin reduced the risks of cardiovascular and kidney events similarly in primary and secondary prevention subgroups of participants with T2D. Canagliflozin reduced the risk of MACE‐3P, cardiovascular death, HHF, and ESKD, with no statistical evidence of treatment effect heterogeneity for primary or secondary prevention subgroups. The magnitude of the absolute reductions assessed for the cardiovascular events with canagliflozin were numerically greater in the secondary prevention subgroup compared with the primary prevention subgroup.

A prior prespecified analysis from the CANVAS Program alone demonstrated that canagliflozin reduced MACE‐3P, cardiovascular, and kidney outcomes, with no statistical evidence of heterogeneity of the treatment effect across the primary and secondary prevention subgroups.^14^ Likewise, a similar analysis in primary and secondary prevention subgroups from the CREDENCE trial showed no treatment effect heterogeneity across cardiovascular and renal outcomes.^15^ A non–participant‐level meta‐analysis focusing on cardiovascular outcomes across primary and secondary prevention subgroups also aligns with our results but did not evaluate kidney outcomes.^17^ SGLT2i appears to have greater risk reduction for cardiovascular events, such as HF in individuals with worsened kidney function.^5^, ^18^, ^19^ UACR and eGFR are established markers for risk of future worsening of kidney function and cardiovascular events, such as HHF. Our analysis extends these prior analyses by using participant‐level data from both the CANVAS Program and CREDENCE trial. The CREDENCE trial enrolled participants with T2D and albuminuric CKD, whereas in the CANVAS Program, 69.8% of participants had normoalbuminuria. The present analysis provides a more demographically diverse population with regard to kidney function and levels of albuminuria to evaluate the impact of canagliflozin in primary and secondary prevention subgroups. The consistency of our results highlights the role of canagliflozin across primary and secondary prevention populations among individuals with a broad distribution of cardiovascular and kidney risk profiles.

Among those with T2D, in addition to MACE‐3P, HHF has emerged as an important clinical event that has historically been ignored by regulators as an important event in clinical trials. The present analysis reinforces the role of canagliflozin in MACE‐3P risk reduction in primary and secondary prevention subgroups. Our results showed consistent treatment effects irrespective of cardiovascular disease history. Although prevalent ASCVD is a major risk factor for the development of HF, even individuals without ASCVD are at increased risk of HF events.^20^ Because ≈60% to 65% of all individuals with T2D do not have clinical atherosclerotic disease, prevention of cardiovascular death or HHF across primary and secondary prevention populations remains an important population health issue. In addition to the CANVAS Program and CREDENCE trial, the DECLARE‐TIMI 58 (Multicenter Trial to Evaluate the Effect of Dapagliflozin on the Incidence of Cardiovascular Events) enrolled participants with T2D with ASCVD (40.6%) or multiple cardiovascular risk factors (59.4%).^21^ The DECLARE‐TIMI 58 demonstrated that dapagliflozin was neutral for the primary efficacy outcome of MACE‐3P (HR, 0.93 [95% CI, 0.84–1.03) without statistical heterogeneity between those with and without ASCVD (*P*_interaction_=0.25). Dapagliflozin did reduce the risk of the second primary efficacy outcome, the composite of cardiovascular death, or HHF (HR, 0.83 [95% CI, 0.73–0.95]). A subgroup analysis demonstrated reductions in the risk of cardiovascular death or HHF in both primary prevention (HR, 0.83 [95% CI, 0.71–0.98]) and secondary prevention (HR, 0.84 [95% CI, 0.67–1.04]; *P*_interaction_=0.99) populations. The effect of sotagliflozin in the SCORED (Cardiovascular and Renal Events in Patients With Type 2 Diabetes and Moderate Renal Impairment Who Are at Cardiovascular Risk) trial demonstrated that dual SGLT1 and 2 inhibitor (SGLT1/2i) reduced the risk of primary outcomes of death from cardiovascular causes and hospitalizations and urgent visits for HF across those with diabetes and CKD, regardless of albuminuria.^22^ Our results align with these analyses demonstrating the usefulness of SGLT2i to reduce the risk of cardiovascular death or HHF across the primary and secondary prevention subgroups.

Prior meta‐analyses, which have included the CANVAS Program and CREDENCE trial, have demonstrated that SGLT2i reduced the risk of kidney outcomes, including progression to ESKD (SGLT2i versus placebo; HR, 0.65 [95% CI, 0.53–0.81]).^5^ Our results expand on this, demonstrating that canagliflozin reduces the risk of kidney outcomes across primary and secondary prevention populations. Globally, of the long‐term T2D‐related complications, CKD imparts some of the highest burden both in terms of burden to health care systems and risk of subsequent morbidity and mortality.^23^ Therefore, prevention of CKD represents an important public health initiative. Biomarker studies from the CANVAS Program demonstrated that, at baseline, higher levels of the inflammatory markers TNFR‐1 (tumor necrosis factor receptor 1) and TNFR‐2 (tumor necrosis factor receptor 2) were associated with increased risk of kidney outcomes among normoalbuminuric participants.^24^, ^25^ These results were independent of the presence of baseline atherosclerotic disease. Furthermore, canagliflozin, compared with placebo, reduced TNFR‐1 and TNFR‐2 over time even after adjustment for baseline ASCVD history. In totality, these data reinforce the kidney benefit of canagliflozin among individuals with T2D regardless of the presence of ASCVD.

Our analysis was limited by the fact that these trials were not designed to discern treatment differences with respect to primary and secondary prevention. However, analysis by primary and secondary prevention subgroups represented a prespecified analysis for the CANVAS Program. In contrast, analysis of the primary and secondary outcomes in the CREDENCE trial was planned for hierarchical testing, with subgroup analyses for the primary outcome prespecified in both subgroups. The increased number of events within our pooled subgroups expanded the prior analysis from the CANVAS Program that evaluated the impact of canagliflozin across primary and secondary subgroups. The inclusion criteria of the CANVAS Program led to a population with higher cardiovascular events, and the inclusion criteria of the CREDENCE trial led to a population at higher risk of progression of diabetic kidney disease; therefore, our analysis may not generalize to real‐world primary prevention populations. Because our primary prevention subgroup was smaller and had a lower risk for cardiovascular events, there may have been limited statistical power to exclude heterogeneity between the primary and secondary prevention subgroups. The mean duration of follow‐up from the CANVAS Program (3.6 years) and CREDENCE trial (2.6 years) may have also limited the ability to determine greater magnitudes of benefit in primary and secondary subgroups.

The CANVAS Program enrolled participants with T2D at high cardiovascular risk and the CREDENCE trial enrolled participants with T2D and albuminuric kidney disease. In this pooled, participant‐level analysis of the CANVAS Program and CREDENCE trial, canagliflozin reduced the risk of MACE‐3P, cardiovascular, and kidney outcomes, without statistical evidence of treatment effect modification between the primary and secondary prevention subgroups. These findings were maintained for the outcomes of cardiovascular death and all‐cause mortality. Future implementation studies are warranted that aim to optimize the use of canagliflozin among eligible individuals as a part of standard of care. These results reinforce the role of canagliflozin in cardiorenal prevention and treatment in individuals with T2D.

## Sources of Funding

Figure preparation support was provided by K. Caldwell of Lumanity Communications Inc., and was funded by Janssen Canada Inc. This study was sponsored by Janssen, Inc. The CANVAS Program and CREDENCE trial were sponsored by Janssen Research & Development, LLC, and were conducted collaboratively by the sponsor, an academic‐led steering committee, and the Academic Research Organization, George Clinical. Canagliflozin has been developed by Janssen Research & Development, LLC, in collaboration with Mitsubishi Tanabe Pharma Corporation.

## Disclosures

Dr Sharma has received support from the FRSQ‐Junior 1 clinician scientist award, the Alberta Innovates Health Solution Clinician Scientist fellowship, the European Society of Cardiology Young Investigator research grant, Janssen, Roche Diagnostics, AstraZeneca, Boehringer Ingelheim, Novartis, Servier, Novo Nordisk, and the Canadian Cardiovascular Society Bayer Vascular award. Dr Levin served as scientific advisor to Boehringer Ingelheim, AstraZeneca, National Institute of Diabetes and Digestive and Kidney Diseases (NIDDK), OccuRx, and Chinook Therapeutics; served on a data safety monitoring board or scientific committee for NIDDK, NIH, Kidney Precision Medicine, CURE consortium, and the University of Washington Kidney Research Institute Scientific Advisory Committee; received research funding from Canadian Institutes of Health Research, Kidney Foundation of Canada, GSK, AstraZeneca, and Boehringer Ingelheim; and received fees for time as CREDENCE national coordinator from Janssen, directed to her academic team. Dr Bajaj reports research support paid to his institution by Amgen, AstraZeneca, Boehringer Ingelheim, Ceapro, Eli Lilly and Company, Gilead, Janssen, Kowa Pharmaceuticals Co. Ltd, Madrigal Pharmaceuticals, Merck, Novartis, Novo Nordisk, Pfizer, Sanofi, and Tricida. Dr Mancini has received support from Janssen, AstraZeneca, Boehringer Ingelheim, Novartis, Novo Nordisk. A. Slee is an employee of New Arch Consulting and received funding from Janssen for this analysis. Dr Ang and W. Rapattoni are employees of Janssen. B.L. Neuen received fees for travel support, advisory boards, scientific presentations, and steering committee roles from AstraZeneca, Bayer, Boehringer Ingelheim, Cambridge Healthcare Research, and Janssen, with all honoraria paid to his institution. C. Arnott received honoraria from Amgen; received support from a National Health and Medical Research Council/Medical Research Future Fund, Priority Fellowship, and an NSW Health EMC grant; and is an employee of the George Institute for Global Health. V. Perkovic received fees for an advisory board, steering committee, or scientific presentation from AbbVie, Amgen, Astellas, AstraZeneca, Bayer, Boehringer Ingelheim, Chinook Therapeutics, CSL Vifor, Gilead, GSK, Janssen, MedImmune, Mitsubishi Tanabe, Mundipharma, Novartis, Novo Nordisk, Otsuka, and Travere Therapeutics, Inc.; received speaker fees from Janssen; served on a data and safety monitoring committee for Dimerix; served on the board of directors for George Clinical, Garvan Institute, George Institute, Victor Chang Cardiac Research Institute, Ingham Institute, and Mindgardens Neuroscience Network; and has stock in George Clinical. Dr Mahaffey has received research grants or contracts that support his research projects from the following companies: AHA, Apple, Inc, Bayer, California Institute Regenerative Medicine, Eidos, Ferring, Gilead, Google (Verily), Idorsia, Johnson & Johnson, Luitpold, Novartis, PAC‐12, Precordior, Sanifit; has provided consulting or other services (including CME) for the following companies (personal income): Amgen, Applied Therapeutics, Bayer, BMS, BridgeBio, CSL Behring, Elsevier, Fibrogen, Fosun Pharma, Johnson & Johnson, Lexicon, Moderna, Myokardia, Novartis, Novo Nordisk, Otsuka, Phasebio, Portola, Quidel, Sanofi, and Theravance; and has equity in the following companies: Precordior and Regencor. The remaining authors have no disclosures to report.

## Supporting information

Tables S1–S2

## References

[1] Tancredi M , Rosengren A , Svensson AM , Kosiborod M , Pivodic A , Gudbjornsdottir S , Wedel H , Clements M , Dahlqvist S , Lind M . Excess mortality among persons with type 2 diabetes. N Engl J Med. 2015;373:1720–1732. doi: 10.1056/NEJMoa1504347 26510021

[2] Morton JI , McDonald SP , Salim A , Liew D , Shaw JE , Magliano DJ . Projecting the incidence of type 2 diabetes‐related end‐stage kidney disease until 2040: a comparison between the effects of diabetes prevention and the effects of diabetes treatment. Diabetes Care. 2021;44:1515–1523. doi: 10.2337/dc21-0220 34024758

[3] Einarson TR , Acs A , Ludwig C , Panton UH . Prevalence of cardiovascular disease in type 2 diabetes: a systematic literature review of scientific evidence from across the world in 2007–2017. Cardiovasc Diabetol. 2018;17:83. doi: 10.1186/s12933-018-0728-6 29884191 PMC5994068

[4] Einarson TR , Acs A , Ludwig C , Panton UH . Economic burden of cardiovascular disease in type 2 diabetes: a systematic review. Value Health. 2018;21:881–890. doi: 10.1016/j.jval.2017.12.019 30005761

[5] Neuen BL , Young T , Heerspink HJL , Neal B , Perkovic V , Billot L , Mahaffey KW , Charytan DM , Wheeler DC , Arnott C , et al. SGLT2 inhibitors for the prevention of kidney failure in patients with type 2 diabetes: a systematic review and meta‐analysis. Lancet Diabetes Endocrinol. 2019;7:845–854. doi: 10.1016/S2213-8587(19)30256-6 31495651

[6] Zelniker TA , Wiviott SD , Raz I , Im K , Goodrich EL , Bonaca MP , Mosenzon O , Kato ET , Cahn A , Furtado RHM , et al. SGLT2 inhibitors for primary and secondary prevention of cardiovascular and renal outcomes in type 2 diabetes: a systematic review and meta‐analysis of cardiovascular outcome trials. Lancet. 2019;393:P31–P39. doi: 10.1016/S0140-6736(18)32590-X 30424892

[7] Sharma A , Pagidipati NJ , Califf RM , McGuire DK , Green JB , Demets D , George JT , Gerstein HC , Hobbs T , Holman RR , et al. Impact of regulatory guidance on evaluating cardiovascular risk of new glucose‐lowering therapies to treat type 2 diabetes mellitus: lessons learned and future directions. Circulation. 2020;141:843–862. doi: 10.1161/CIRCULATIONAHA.119.041022 31992065

[8] Mancini GBJ , O'Meara E , Zieroth S , Bernier M , Cheng AYY , Cherney DZI , Connelly KA , Ezekowitz J , Goldenberg RM , Leiter LA , et al. 2022 Canadian Cardiovascular Society guideline for use of GLP‐1 receptor agonists and SGLT2 inhibitors for cardiorenal risk reduction in adults. Can J Cardiol. 2022;38:1153–1167. doi: 10.1016/j.cjca.2022.04.029 35961754

[9] Sharma A , Butler J , Zieroth S , Giannetti N , Verma S . Treatment of heart failure with sodium glucose co‐transporter‐2 inhibitors in people with type 2 diabetes mellitus: current evidence and future directions. Diabet Med. 2019;36:1550–1561. doi: 10.1111/dme.14140 31536660

[10] Neal B , Perkovic V , Mahaffey KW , de Zeeuw D , Fulcher G , Erondu N , Shaw W , Law G , Desai M , Matthews D , et al. Canagliflozin and cardiovascular and renal events in type 2 diabetes. N Engl J Med. 2017;377:644–657. doi: 10.1056/NEJMoa1611925 28605608

[11] Perkovic V , Jardine MJ , Neal B , Bompoint S , Heerspink HJL , Charytan DM , Edwards R , Agarwal R , Bakris G , Bull S , et al. Canagliflozin and renal outcomes in type 2 diabetes and nephropathy. N Engl J Med. 2019;380:2295–2306. doi: 10.1056/NEJMoa1811744 30990260

[12] Eberly LA , Yang L , Eneanya ND , Essien U , Julien H , Nathan AS , Khatana SAM , Dayoub EJ , Fanaroff AC , Giri J , et al. Association of race/ethnicity, gender, and socioeconomic status with sodium‐glucose cotransporter 2 inhibitor use among patients with diabetes in the US. JAMA Netw Open. 2021;4:e216139. doi: 10.1001/jamanetworkopen.2021.6139 33856475 PMC8050743

[13] Sharma A , Aziz H , Verma S , Abramson BL , Choi R , Chua GL , Connelly KA , Honos G , Mancini GBJ , Ramer SA , et al. Permission to prescribe: do cardiologists need permission to prescribe diabetes medications that afford cardiovascular benefit? Curr Opin Cardiol. 2021;36:672–681. doi: 10.1097/HCO.0000000000000892 34173772

[14] Mahaffey KW , Neal B , Perkovic V , de Zeeuw D , Fulcher G , Erondu N , Shaw W , Fabbrini E , Sun T , Li Q , et al. Canagliflozin for primary and secondary prevention of cardiovascular events: results from the CANVAS program. Circulation. 2018;137:323–334. doi: 10.1161/CIRCULATIONAHA.117.032038 29133604 PMC5777572

[15] Mahaffey KW , Jardine MJ , Bompoint S , Cannon CP , Neal B , Heerspink HJL , Charytan DM , Edwards R , Agarwal R , Bakris G , et al. Canagliflozin and cardiovascular and renal outcomes in type 2 diabetes and chronic kidney disease in primary and secondary cardiovascular prevention groups. Circulation. 2019;140:739–750. doi: 10.1161/CIRCULATIONAHA.119.042007 31291786 PMC6727954

[16] Neal B , Perkovic V , Matthews DR , Mahaffey KW , Fulcher G , Meininger G , Erondu N , Desai M , Shaw W , Vercruysse F , et al. Rationale, design and baseline characteristics of the CANagliflozin cardioVascular assessment study‐renal (CANVAS‐R): a randomized, placebo‐controlled trial. Diabetes Obes Metab. 2017;19:387–393. doi: 10.1111/dom.12829 28120497 PMC5348724

[17] Arnott C , Li Q , Kang A , Neuen BL , Bompoint S , Lam CSP , Rodgers A , Mahaffey KW , Cannon CP , Perkovic V , et al. Sodium‐glucose cotransporter 2 inhibition for the prevention of cardiovascular events in patients with type 2 diabetes mellitus: a systematic review and meta‐analysis. J Am Heart Assoc. 2020;9:e014908. doi: 10.1161/JAHA.119.014908 31992158 PMC7033896

[18] McGuire DK , Shih WJ , Cosentino F , Charbonnel B , Cherney DZI , Dagogo‐Jack S , Pratley R , Greenberg M , Wang S , Huyck S , et al. Association of SGLT2 inhibitors with cardiovascular and kidney outcomes in patients with type 2 diabetes: a meta‐analysis. JAMA Cardiol. 2021;6:148–158. doi: 10.1001/jamacardio.2020.4511 33031522 PMC7542529

[19] Toyama T , Neuen BL , Jun M , Ohkuma T , Neal B , Jardine MJ , Heerspink HL , Wong MG , Ninomiya T , Wada T , et al. Effect of SGLT2 inhibitors on cardiovascular, renal and safety outcomes in patients with type 2 diabetes mellitus and chronic kidney disease: a systematic review and meta‐analysis. Diabetes Obes Metab. 2019;21:1237–1250. doi: 10.1111/dom.13648 30697905

[20] Sacre JW , Magliano DJ , Shaw JE . Incidence of hospitalization for heart failure relative to major atherosclerotic events in type 2 diabetes: a meta‐analysis of cardiovascular outcomes trials. Diabetes Care. 2020;43:2614–2623. doi: 10.2337/dc20-0654 32958618

[21] Wiviott SD , Raz I , Bonaca MP , Mosenzon O , Kato ET , Cahn A , Silverman MG , Zelniker TA , Kuder JF , Murphy SA , et al. Dapagliflozin and cardiovascular outcomes in type 2 diabetes. N Engl J Med. 2019;380:347–357. doi: 10.1056/NEJMoa1812389 30415602

[22] Bhatt DL , Szarek M , Pitt B , Cannon CP , Leiter LA , McGuire DK , Lewis JB , Riddle MC , Inzucchi SE , Kosiborod MN , et al. Sotagliflozin in patients with diabetes and chronic kidney disease. N Engl J Med. 2021;384:129–139. doi: 10.1056/NEJMoa2030186 33200891

[23] DeFronzo RA , Reeves WB , Awad AS . Pathophysiology of diabetic kidney disease: impact of SGLT2 inhibitors. Nat Rev Nephrol. 2021;17:319–334. doi: 10.1038/s41581-021-00393-8 33547417

[24] Waijer SW , Sen T , Arnott C , Neal B , Kosterink JGW , Mahaffey KW , Parikh CR , de Zeeuw D , Perkovic V , Neuen BL , et al. Association between TNF receptors and KIM‐1 with kidney outcomes in early‐stage diabetic kidney disease. Clin J Am Soc Nephrol. 2022;17:251–259. doi: 10.2215/CJN.08780621 34876454 PMC8823939

[25] Sen T , Li J , Neuen BL , Neal B , Arnott C , Parikh CR , Coca SG , Perkovic V , Mahaffey KW , Yavin Y , et al. Effects of the SGLT2 inhibitor canagliflozin on plasma biomarkers TNFR‐1, TNFR‐2 and KIM‐1 in the CANVAS trial. Diabetologia. 2021;64:2147–2158. doi: 10.1007/s00125-021-05512-5 34415356 PMC8423682

