# Supplementary material for: Primary and Secondary Cardiovascular and Kidney Prevention With Canagliflozin: Insights From the CANVAS Program and CREDENCE Trial
Source: J Am Heart Assoc. 2024 Jan 19;13(3):e031586. doi: 10.1161/JAHA.123.031586 (PMC11056176; doi:10.1161/JAHA.123.031586)
Supplement: Supplementary file 1 — Tables S1–S2 [file JAH3-13-e031586-s001.pdf]

SUPPLEMENTAL MATERIAL

Table S1. Proportion of each adjusted covariate in the fitted event rate values

	Overall	Primary	Secondary
Estimated glomerular filtration rate ≥ 60 (mL/min/1.73 m^2)	67.8%	67.2%	68.2%
Urine albumin/creatinine ratio ≤ 300 mg/g (33.9mg/mmol)	67.9%	61.8%	71.8%
Duration of diabetes <13 years	50.6%	48.2%	52.2%
Male	64.8%	58.2%	68.9%

Table S2. Crude and adjusted event rates values fitted at means of the primary and secondary prevention groups

			Event Rate/1,000 PYAR		Treatment Comparison			
Endpoint	Group	Treatment	Crude	Adjusted	Difference	95% CI		P-value
Time to 1st MACE	Primary	Cana	17.2	17.8	2.83	-1.16	6.82	0.165
		Placebo	21.6	20.6				
	Secondary	Cana	37.9	38.7	6.89	1.95	11.82	0.006
		Placebo	47.4	45.7				
Time to CV death	Primary	Cana	7.9	8.1	0.82	-1.73	3.37	0.531
		Placebo	9.8	8.9				
	Secondary	Cana	16.6	16.8	2.19	-0.83	5.21	0.155
		Placebo	20.6	19.1				
Time to 1st Non-Fatal MI	Primary	Cana	5.4	5.5	0.75	-1.45	2.94	0.506
		Placebo	6.4	6.2				
	Secondary	Cana	13.8	13.7	3.00	0.02	5.99	0.048
		Placebo	17.3	16.8				
Time to 1st Non-Fatal Stroke	Primary	Cana	4.5	4.6	1.61	-0.52	3.74	0.139
		Placebo	6.4	6.2				
	Secondary	Cana	9.6	9.9	1.33	-1.11	3.77	0.285
		Placebo	11.5	11.2				
Time to 1st Hospitalized Heart Failure	Primary	Cana	4.6	4.3	2.73	0.70	4.76	0.008
		Placebo	9.0	7.1				
	Secondary	Cana	9.6	8.6	4.57	2.23	6.92	0.000
		Placebo	16.8	13.2				
Time to 1st Hospitalized Heart Failure or CV Death	Primary	Cana	12.3	12.3	2.80	-0.45	6.05	0.091
		Placebo	17.3	15.1				
	Secondary	Cana	24.2	23.3	7.64	3.82	11.46	<.0001
		Placebo	35.6	30.9				
Time to All-Cause Death	Primary	Cana	13.6	13.9	1.81	-1.58	5.21	0.296
		Placebo	17.0	15.7				
	Secondary	Cana	23.9	23.6	2.77	-0.87	6.41	0.136
		Placebo	28.8	26.4				
Time to Doubling SC	Primary	Cana	6.1	3.2	2.33	1.03	3.63	0.000
		Placebo	12.2	5.5				
	Secondary	Cana	6.7	1.8	1.14	0.39	1.89	0.003
		Placebo	6.6	2.9				
Time to ESKD	Primary	Cana	8.4	1.3	0.58	0.06	1.11	0.030
		Placebo	15.3	1.9				
	Secondary	Cana	4.1	0.6	0.29	0.01	0.57	0.045
		Placebo	5.5	0.9				
	Primary	Cana	3.4	3.8	2.12	0.82	3.42	0.001

Time to ESKD or Doubling SC		Placebo	17.3	5.9				
	Secondary	Cana	8.7	2.2	1.11	0.32	1.89	0.006
		Placebo	10.7	3.3				

CV, Cardiovascular; MACE , Major Adverse CV events; MI, Myocardial Infarction; PYAR, Patient Years Adjusted Rates; SC serum creatinine
